# Supplementary material for: Knowledge of Health Professionals Regarding Vegetarian Diets from Pregnancy to Adolescence: An Observational Study
Source: Nutrients. 2019 May 23;11(5):1149. doi: 10.3390/nu11051149 (PMC6566389; doi:10.3390/nu11051149)
Supplement: Supplementary file 1 [file nutrients-11-01149-s001.pdf]

SOCIODEMOGRAPHIC CHARACTERISTICS

Indicate your professional profile even if you do not intend to give your consent

I don't agree Nurse Paediatric nurse Midwife Staff nurse Health care support workers

Gender

Female Male

Nationality

Italian

Non-Italian

Age (years)

20-24 35-39 50-54

25-29 40-44 > 55

30-34 45-49

Education

High school diploma Degree

Work experience (years)

0-4 15-19 30-34

5-9 20-24 35-39

10-14 25-29 >39

Work experience in the maternal-child area

Obstetrics Neonatal Paediatric Community service

Have you participated in a developmental nutrition course in the last 5 years?

Yes No

Do parents ask you for information about their child's feeding or diet?

Never Rarely Often Always

Please indicate with an X which foods are part of your diet and how often you consume them

	More than once a week	Every 15 days	More than once a month
**Red meat**			
**Poultry**			
**Fish**			
**Dairy products**			
**Eggs**			

Do you consume honey?

Yes No

Do you have paediatric-age children?

Yes No

If you have children, indicate with an X which foods are part of their diet and how often they consume them

	More than once a week	Every 15 days	More than once a month
**Red meat**			
**Poultry**			
**Fish**			
**Dairy products**			
**Eggs**			

KNOWLEDGE

1. Definition of a vegetarian diet:

Multiple answers may be chosen

Exclusion of meat and fish

Exclusion of meat, inclusion of eggs and dairy products

Inclusion of eggs, dairy products, and small amounts of fish or poultry

Exclusion of meat, fish and poultry, inclusion of dairy products and eggs

Exclusion of meat, fish, poultry and eggs, inclusion of dairy products
Exclusion of meat, fish, poultry and dairy products, inclusion of eggs
Don't know

2. Definition of a vegan diet:

Mark only one answer

Exclusion of meat, fish and eggs, inclusion of dairy products
Exclusion of meat and fish, inclusion of honey
Exclusion of meat, fish, eggs and dairy products, inclusion of honey
Exclusion of meat, fish and poultry as well as eggs, dairy products and honey
Don't know

3. An animal-based diet provides more health benefits than a plant-based diet

True False Don't know

4. A 100% plant diet, compared to the intake of a mainly-plant diet, does not further reduce cardiovascular risk factors or the development of diabetes

True False Don't know

5. Many chronic diseases, such as cardiovascular diseases, type 2 diabetes, hypertension and some types of cancer, are

More frequent in omnivores than in vegetarians
More frequent in vegetarians than in omnivores
Equally prevalent in omnivores and vegetarians
Don't know

6. A planned vegetarian diet is nutritionally adequate during pregnancy, breastfeeding, the first and second phases of childhood, and adolescence

True False Don't know

7. There is clear proof that the first 1000 days of life (from conception until the age of 2 years) are particularly critical to good development and healthy growth. A plant-based diet is able to satisfy the nutritional demands of an infant in the first 1000 days of life.

True False Don't know

8. A vegetarian/vegan mother is at greater risk of deficiency in

Mark only one answer

Vitamin D
Vitamin B12
Vitamin A
Don't know

9. Planned vegetarian and vegan diets during pregnancy involve higher risks of pregnancy difficulties or birth defects

True False Don't know

10. The lacto-ovo vegetarian mother can breastfeed without supplementation

True False Don't know

11. The vegan mother can breastfeed without supplementation

True False Don't know

12. Children who follow a planned vegetarian diet have an adequate energy intake similar to that of omnivorous children

True False Don't know

13. During complementary feeding, the infant must receive a sufficient amount of

Mark only one answer

Vitamin B12
Vitamin B12, iron, zinc and folate
Vitamin B12, Vitamin D, iron, zinc, folate, n-3 LCPUFA, protein and calcium
Don't know

14. A vegetarian or 100% plant diet represents a protective factor against the development of infant overweight

True False Don’t know

15. What supplemental nutrient should not be missing in the diet of a vegetarian or vegan child?

Mark only one answer

Iron
Calcium
Zinc
Vitamin B12
Don’t know

16. A teenager who chooses a vegetarian diet is

Multiple answers may be selected

Potentially at risk of impulsive choices
Potentially at risk of making ill-informed choices
Potentially at risk for an eating disorder
Don’t know

17. Plant proteins have less bioavailability than animal proteins

True False Don’t know

18. In the first 2 years of life, the amount of fibre in the diet must be limited

True False Don’t know

19. Iron is a critical nutrient only for vegetarian/vegan children under 2 years of age

True False Don’t know

20. The sources that meet the calcium requirements are

Multiple answers may be selected

Breast milk
Formula
Increased fibre
Low-calcium salt water
Don’t know

21. Zinc has the following characteristics

Mark only one answer

Greater bioavailability in vegetarian diets
Greater bioavailability in omnivorous diets
Equal bioavailability in vegetarian diets and omnivorous diets
Don’t know

22. Vitamin D levels appear to be influenced by diet and are lower in vegetarian children

True False Don’t know

23. Long-chain omega-3 fatty acid levels are

Make only one answer

Lower in vegetarians and vegans
Similar to those of omnivores for vegetarians and lower in vegans
Lower in vegetarians, typically absent in vegans
Don’t know
